# Treatment of irritable bowel syndrome with a novel colonic irrigation system: a pilot study

**DOI:** 10.1007/s10151-016-1491-x

**Published:** 2016-05-19

**Authors:** H.-H. Hsu, W.-H. Leung, G.-C. Hu

**Affiliations:** 1 Division of Colorectal Surgery, Department of Surgery, Mackay Memorial Hospital, 92, Sec. 2, Zhongshan N. Rd., Taipei City, 10449 Taiwan; 2 Department of Rehabilitation Medicine, Mackay Memorial Hospital, Taipei City, Taiwan

**Keywords:** Ashong colonic irrigation apparatus, Colonic irrigation, Irritable bowel syndrome

## Abstract

**Background:**

Medical treatments for irritable bowel syndrome (IBS) are often disappointing. A colonic irrigation system, the Ashong colonic irrigation apparatus (ACIA), was designed as a patient-administered device for defecation disorders. This pilot study evaluated the efficacy and safety of ACIA for IBS.

**Methods:**

Eighteen patients, 12 with constipation-dominant IBS (IBS-C) and 6 with diarrhea-dominant IBS (IBS-D) group, were studied. Patients were randomized into treatments of 1–4 weeks. Colonic irrigation was performed twice daily for 6 consecutive days per week. To determine the response to treatment, bowel movement frequency, stool consistency, abdominal pain, patient satisfaction with bowel movements, and distress/discomfort due to symptoms were assessed.

**Results:**

The scores of abdominal pain (*p* < 0.001), satisfaction (*p* < 0.001), and distress/discomfort (*p* < 0.001) improved significantly. The frequency of bowel movements in the IBS-C group increased from 1.68 to 3.78 times per week (*p* < 0.001). The occurrence of Bristol Stool Scale type 1 and 2 stool passage decreased from 45 to 13 % (*p* = 0.009) in the IBS-C group and type 6 and 7 stools decreased from 62 to 28 % (*p* = 0.005) in the IBS-D group. Only mild adverse events occurred, and all patients completed treatment.

**Conclusions:**

Colonic irrigation with ACIA is safe and can improve abdominal pain, constipation, and diarrhea associated with IBS. Patients were more satisfied with their bowel movements and found their symptoms were less disturbing. Larger studies on long-term efficacy and quality of life and on placebo effects are needed.

## Introduction

Irritable bowel syndrome (IBS) is a functional gastrointestinal disorder according to the Rome III classification [1]. IBS affects 10–20 % of the population, predominantly females. The pathogenesis of IBS involves abnormalities in motility, visceral sensation, brain–gut interaction, and psychosocial distress. More recently, altered gut immune activation, intestinal permeability, and the intestinal and colonic microbiome have been shown to contribute to IBS. Treatment of IBS includes psychological support, exercises, diet management, and medical treatments. The symptoms of constipation-dominant IBS (IBS-C) may be improved by fiber supplements, laxatives, and avoidance of short-chain carbohydrates and gluten. Anti-diarrheal medications, 5-HT3 antagonists, antispasmodics, and antidepressants are also effective [2]. A recent meta-analysis showed that administration of probiotics also improved symptoms and quality of life (QoL) for patients with IBS [3].

Rectal or colonic irrigation has been shown to be successful in treating neurogenic, congenital, and idiopathic bowel dysfunction; constipation; and fecal incontinence, as well as improving QoL [4–10]. Patients with fecal incontinence after low anterior resection may also benefit from colonic irrigation [11]. Medical treatments are often temporary and disappointing. The role of colonic irrigation for IBS, however, is not yet well established.

**Fig. 1:**
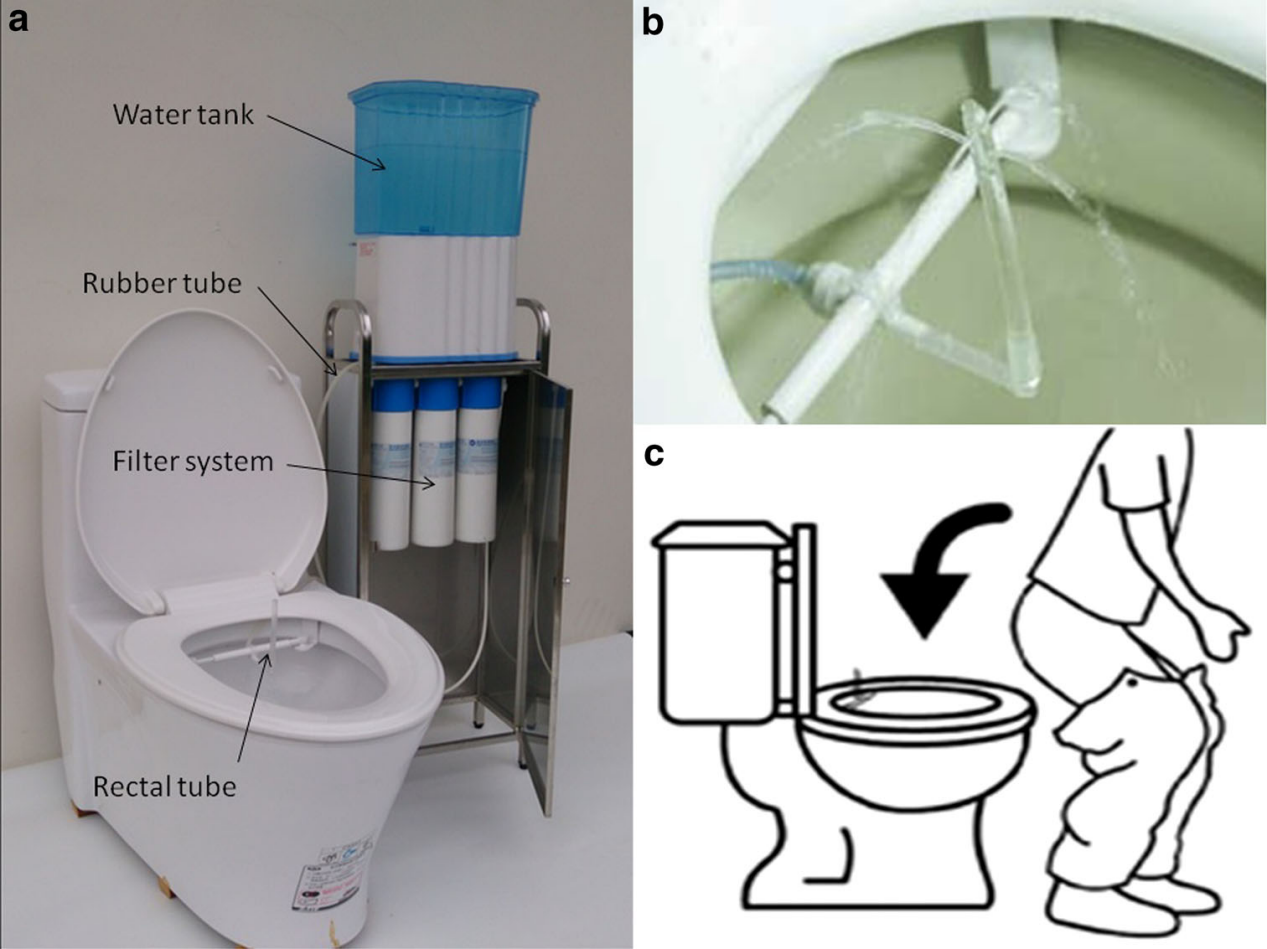
**a** Overall view of the Ashong colonic irrigation apparatus. Tap water in the water tank flows through the filter system, then passes through the rubber tube, and finally reaches the rectal tube. **b** The rectal tube is fixed to the toilet. Filtered water flows out of the tip of the rectal tube in four directions, and the flow rate is maintained at 5 mL/s. **c** Diagram showing the method of rectal tube insertion

The Ashong colonic irrigation apparatus (ACIA) is a colonic irrigation system approved by the Taiwan Food and Drug Administration. It is a simple device designed for patient self-administration. The device is composed of a water tank, filter system, and an L-shaped disposable soft silicone rectal tube affixed to the toilet (Fig. 1). The water flow rate is maintained at 5 mL per second with a constant water temperature of 36 ± 2 °C. The duration of water flow can be adjusted as required. After sitting properly on the toilet and inserting the rectal tube, patient manipulates the ACIA via a remote control. The aim of this pilot study was to evaluate the efficacy and safety of AICA in the treatment of IBS.

## Materials and methods

### Patient selection

This clinical trial was approved by the Institutional Review Board of Mackay Memorial Hospital. Male and female IBS patients between 20 and 65 years of age who were refractory to previous medicinal treatments were invited for primary screening. Informed consent was obtained from all patients before the screening phase. Exclusion criteria included hernia, inflammatory bowel disease, grade 3 and 4 hemorrhoids, anal fissure, rectal prolapse, megacolon, peptic ulcer disease, previous abdominal surgery, renal impairment, uncontrolled hypertension, pregnancy, paralysis, and aortic aneurysm. All patients were assessed by double-contrast barium enema or colonoscopy to exclude any organic colonic lesions. Only those who met the Rome III diagnostic criteria of diarrhea-dominant IBS (IBS-D) and IBS-C [1] were included in the study.

### Treatment strategy

ACIA was used with a constant water temperature of 37 °C, water flow at 5 mL/s for 100 s. Colonic irrigation was repeated for 8 cycles to constitute one complete therapeutic section. Patients were allowed to defecate at any time while irrigation was in progress.

Treatments were divided into 1-, 2-, 3- and 4-week courses for IBS-C (subgroups: 1C, 2C, 3C, 4C) and IBS-D (subgroups: 1D, 2D, 3D, 4D) groups in order to evaluate the influence of treatment duration on efficacy. Patients were randomized consecutively, beginning with the 4-week course and continuing through the 1-week course, into each of the 8 treatment subgroups with the aim of producing 8 groups of 3 patients each. Patients received colonic irrigation twice daily for 6 consecutive days per week and were allowed to withdraw from the study at any time. A study nurse stayed beside the patients during irrigation, giving instructions, recording adverse effects, and monitoring safety. After receiving a thorough explanation of the irrigation system, patients were instructed to manipulate the device themselves throughout the study period.

### Patient diary

Patients maintained a record of bowel movement (BM) frequency, stool consistency, and abdominal pain intensity, from 7 days before treatment (pre-treatment phase) to 7 days following treatment (post-treatment phase). Stool consistency was recorded according to Bristol Stool Scale (BSS) (types 1–7) [12]. The scores of abdominal pain, satisfaction with BM, and distress/discomfort due to symptoms were recorded on a 0-to-10 rating scale. A score of 10 indicated the most severe abdominal pain, greatest satisfaction with BM, and least distress/discomfort due to symptoms.

### Evaluation of efficacy and safety

Efficacy was evaluated by measuring treatment-related changes in BM frequency, stool consistency, abdominal pain, satisfaction with BM, and distress/discomfort due to symptoms. All adverse events occurring in the course of the study period were recorded. A mild adverse event was defined as any mild symptom requiring neither medical intervention nor discontinuation of treatment. A moderate adverse event was defined as any intolerable symptom requiring medical intervention and terminating irrigation. A severe adverse event was defined as any life endangering event requiring immediate medical intervention. Patients were provided 24-h access to telephone assistance. Blood serum samples were obtained prior to treatment and 2 days (±1 day) before the end of treatment for analysis of glutamic-oxaloacetate transaminase (GOT), glutamic-pyruvic transaminase (GPT), gamma-glutamyl transpeptidase (γGT), alkaline phosphatase (ALKP), Na^**+**^, K^**+**^, Cl^−^, and blood urea nitrogen (BUN).

### Statistical analysis

Demographic data and clinical characteristics were expressed as proportions for categorical variables and as mean ± standard deviation (SD) values for continuous variables. The paired *t* test or Wilcoxon signed-rank test was used to compare pre- and post-treatment changes in scores of abdominal pain, satisfaction with BM, and distress/discomfort due to symptoms. To assess the pre- and post-treatment changes in BM frequency and stool consistency, participants were enrolled in IBS-C and IBS-D subgroups. Differences between each of the variables were assessed using the two-tailed paired *t* test or *χ*^2^ test, and values of *p* < 0.05 were considered statistically significant. Analyses were carried out using SAS, version 9.0 (SAS, Cary, NC, USA).

## Results

**Fig. 2:**
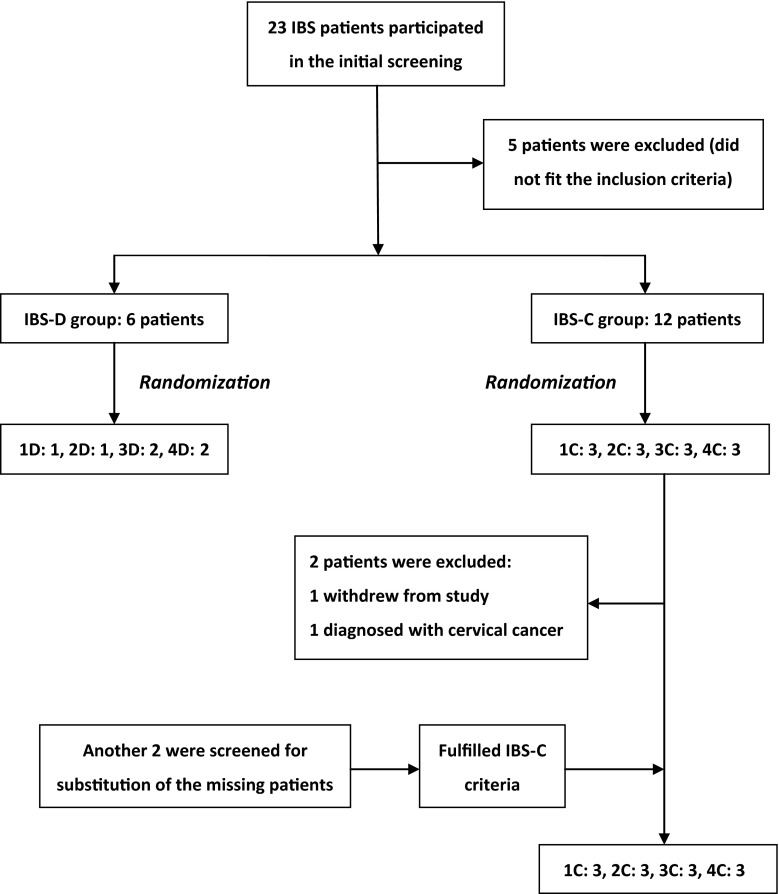
Schematic flow chart from screening to randomization of participants

Between December 18, 2013, and July 18, 2014, 23 patients participated in the initial screening, and only 18 patients fulfilled the diagnostic criteria. One withdrew from the ongoing study, and 1 was excluded due to a diagnosis of cervical cancer. Two additional individuals were screened as potential substitutes for the missing 2 within the same study period, but unfortunately, only 6 patients were enrolled in the IBS-D group. Eighteen patients (13 females; mean age 45.2 (24–62) years) completed the treatments and entered the final analysis (Fig. 2). The IBS-C group was female dominant, whereas the IBS-D group was male dominant (Table 1).

**Table 1:** Demographics of study participants

Group	All	IBS-C	IBS-D
Sex			
Male	5 (28 %)	1 (8.3 %)	4 (67 %)
Female	13 (72 %)	11 (91.7 %)	2 (33 %)
Age (years)			
Mean ± SD	45.2 ± 13.6	44.7 ± 15.1	46.0 ± 11.2
Range	24–62	24–62	31–58

*IBS-C* constipation-dominant irritable bowel syndrome, *IBS-D* diarrhea-dominant irritable bowel syndrome, *SD* standard deviation

**Fig. 3:**
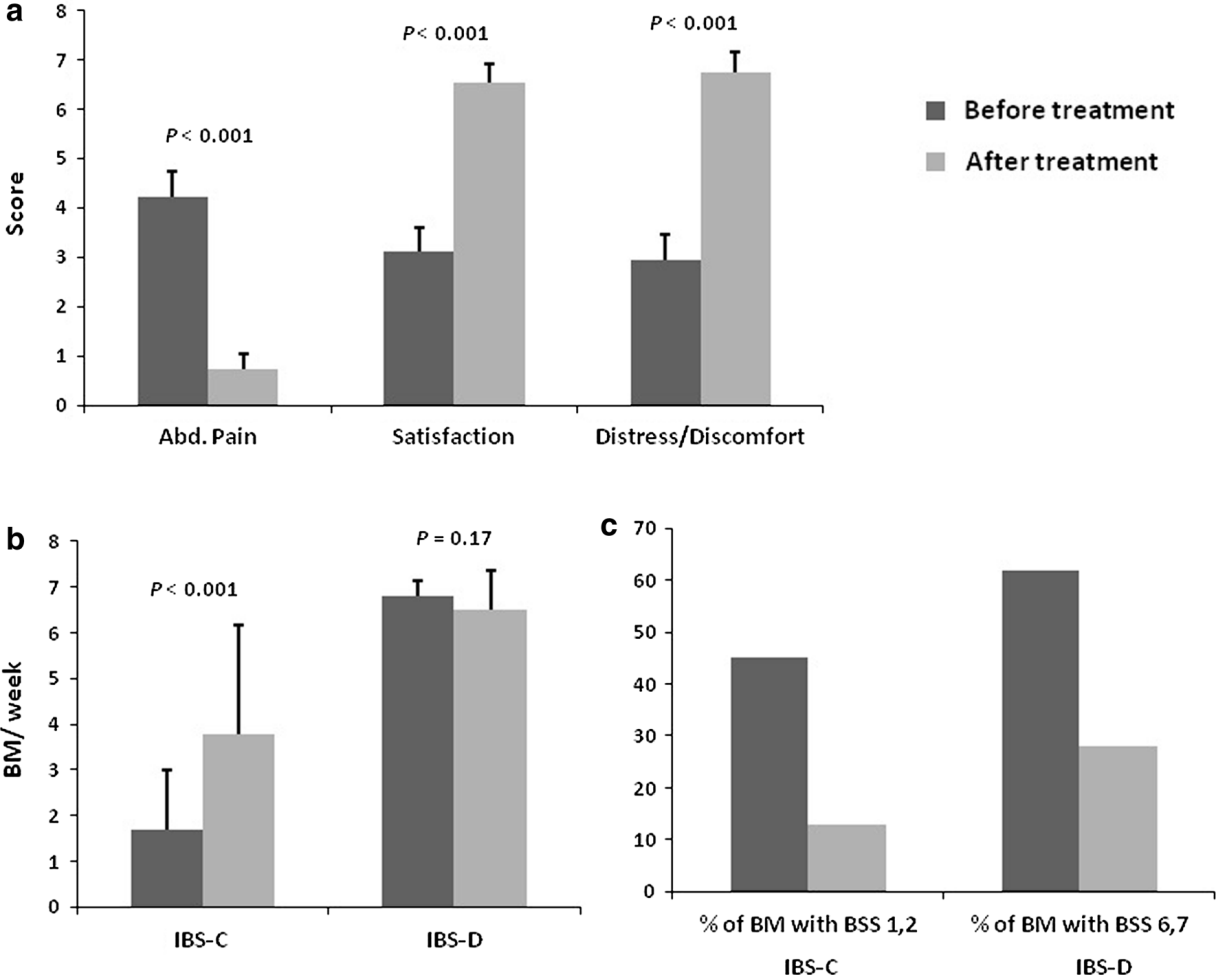
**a** Scores of abdominal pain, satisfaction, and degree of distress/discomfort due to symptoms before and after treatment. Calculations involved all constipation-dominant irritable bowel syndrome and diarrhea-dominant irritable bowel syndrome patients. **b** Frequency of bowel movements before and after treatment. **c** Stool consistency changes in response to colonic irrigation

Overall abdominal pain score decreased significantly from 4.23 to 0.73 (*p* < 0.001) after colonic irrigation treatment. The score of satisfaction with BM increased from 3.10 to 6.52 (*p* < 0.001), and the score of distress/discomfort due to symptoms improved from 2.95 to 6.75 (*p* < 0,001) (Fig. 3a). In addition, the results of the 2 individual groups showed significant improvements. After ACIA treatment, all patients in the IBS-D group and 5 of 12 patients in the IBS-C group experienced complete relief from abdominal pain.

BM frequency in the IBS-C group increased from 1.68 to 3.78 times per week (*p* < 0.001; Fig. 3b), and the percentage of BSS type 1 and 2 stool passage decreased from 45 to 13 % (*p* = 0.009; Fig. 3c) after treatment. Only 1 patient in the 1C subgroup showed no improvement in BM frequency, but still showed an improvement in stool consistency from BSS type 1 to type 4. Even though there was no significant improvement in BM frequency in the IBS-D group (Fig. 3b), the percentage of BSS type 6 and 7 stool passage decreased significantly from 62 to 28 % (*p* = 0.005; Fig. 3c) after colonic irrigation. The only patient in the 1D subgroup showed persistent passage of loose stools, and this was the only patient from the IBS-D group showing no improvement in stool consistency.

One-third of patients experienced mild adverse events with neither discontinuation of treatment nor medical intervention. Abdominal cramping pain occurred in 2 patients (11.1 %), 4 (22.2 %) suffered from a sensation of mild abdominal fullness, 1 patient (5.56 %) experienced general weakness, and 1 presented with anal pain (5.56 %). Only 2 patients suffered from more than one adverse event.

Serum levels for the electrolytes Na^**+**^, K^**+**^, Cl^**−**^, before and after treatment, were all within normal ranges. Lower than normal limits of serum GPT, GOT, γGT, and BUN levels were observed before treatment in 8 participants (44.4 %). One male who was a heavy drinker had elevated basal and post-treatment levels of GOT and GPT.

## Discussion

The beneficial effect of colonic or rectal irrigation on neurogenic or congenital defecation disorders is well known, and functional constipation and diarrhea have also been shown to benefit from rectal irrigation [13]. Even though the exact mechanism underlying the response of IBS to colonic irrigation is not known, our results demonstrate its potential benefit as an IBS treatment modality. A recent animal study by Zhang et al. [14] recognized methylglyoxal as an important cause of IBS symptoms. This bacterial product of anaerobic glycolysis in the large intestine elicits symptoms such as arrhythmia, headache, and diarrhea. Activation of NMDA receptors by methylglyoxol promotes visceral hypersensitivity, resulting in increased 5HT secretion and peristalsis contributing to diarrhea. Visceral hypersensitivity reduces the threshold to noxious stimuli and bowel distension, providing a reasonable explanation for abdominal pain [15, 16], the major feature of IBS. We deduced that methylglyoxol and perhaps other metabolites could be washed out by water irrigation, and reverse visceral hypersensitivity. Zhang’s study may serve to renew interest in the concept “body detoxification” by colonic irrigation, which is at present not well accepted by the medical professions [17].

Almost all participants with IBS-C experienced more than one additional BM per week than before treatment. Colonic irrigation also improved the stool consistency in most participants, whether this was for hard, lumpy stool in IBS-C or loose, watery stool in IBS-D patients. The results demonstrate colonic irrigation is effective in improving IBS-related constipation and diarrhea.

The influence of treatment duration on efficacy was investigated by dividing the treatment into 1- to 4-week courses. Abdominal pain, satisfaction with BM, and distress/discomfort due to symptoms were improved after initiation of colonic irrigation. This improvement was maintained throughout the treatment phase and even up to the post-treatment phase in every subgroup (data not shown). The treatment effects seemed to be independent of the treatment duration, but our short-term data are not adequate to support this conclusion.

Safety is always the greatest concern with bowel irrigation therapy, and rectal perforations and sepsis are not uncommon. Such complications have been attributed to malpractice by patients and non-medical personnel [18–21]. However, the estimated rate of bowel perforation resulting from transanal irrigation is low, with a reported rate of <0.002 % [5, 7]. A well-devised treatment protocol used by experienced practitioners might reduce the risk of bowel perforation [20, 21]. Conversely, undiscovered conditions such as stercoral ulcer [19] or tumors could increase the risk of perforation. In this study, we provided complete colonic evaluations and detailed instructions for the treatment. Our patients experienced only mild adverse events such as sensation of abdominal fullness, weakness, and anal pain which did not necessitate either discontinuation of treatment or medical intervention.

A series of 36 cases of amebiasis cross-infection with 10 colectomies, in which 6 patients at a chiropractic clinic in Western Colorado died, was previously reported [22]. The outbreak led to the use of disposable, single-use parts of colonic irrigation devices [18], and patient-specific colonic irrigation system for defecation disorders are necessary to prevent cross-contamination.

Eight liters of water was required for a complete 1-day treatment, and water intoxication is an important safety concern in colonic irrigation. However, no neurological symptoms or altered serum biochemistry parameters, such as hyponatremia were observed in this study. In addition, since patients were allowed to defecate at any time during irrigation, the amount of water absorption via the colonic mucosa was very small. Lower than normal limit basal serum GOT, GPT, γGT, and BUN levels were observed in about 45 % of the participants, suggesting these could be clinical signs of IBS. Moreover, colonic irrigation did not cause impairment in blood serum biochemistry or electrolyte imbalance. Chronic consumption of alcohol appeared to account for the only candidate with impaired basal and post-treatment GOT and GPT levels. Sacral nerve stimulation and the Malone antegrade continence enema procedure have been shown to be effective in patients with unremitting constipation and defecation disorders, improving QoL [23–26]. However, those are invasive procedures that come with a risk of surgical complications. Rectal or colonic irrigation is considered to be less invasive and effective as well [7, 8]. Irrigation treatment should be considered as the treatment of choice prior to surgical interventions [4, 13]. The concept behind ACIA is no different from that behind the ordinary colonic irrigation sets [4, 7–9], but ACIA is simpler and more convenient.

Our results demonstrated that colonic irrigation with ACIA can be effective for IBS treatment. The improvement of abdominal pain, satisfaction with BM, and distress/discomfort due to symptoms indicated there was good relief of IBS-related problems and the patients were satisfied with the treatment. However, a placebo effect [27] should be taken into consideration. The positive effects on BM frequency and stool consistency are encouraging, and those are objectively measurable. QoL is always an important parameter for the effectiveness evaluation of IBS treatment. In this study, we focused on the feasibility of colonic irrigation on IBS treatment and QoL evaluation is therefore lacking.

The limitations of this study are the small sample size and short follow-up time.

## Conclusions

The Ashong colonic irrigation apparatus is a simple, safe device that allows for patient self-administration of colonic irrigation. Our pilot study showed colonic irrigation can be effective in improving the abdominal pain, constipation, and diarrhea associated with IBS. However, the impact of placebo should be considered and is worth investigation. Larger studies with long-term follow-up are needed in order to draw more reliable conclusions.
